# Associations between Plasma Antibody Levels against *Porphyromonas gingivalis* and Atrial Fibrillation among Community-Dwelling Older Individuals in Japan: A Cross-Sectional Study

**DOI:** 10.3290/j.ohpd.b4528813

**Published:** 2023-10-20

**Authors:** Takashi Hoshino, Noboru Kaneko, Akihiro Yoshihara, Masanori Iwasaki, Kana Suwama, Yumi Ito, Junta Tanaka, Ichiei Narita, Hiroshi Ogawa

**Affiliations:** a Graduate Student, Division of Preventive Dentistry, Faculty of Dentistry & Graduate School of Medical and Dental Sciences, Niigata University, Niigata, Japan. Conceptualization, formal analysis, investigation, data curation, drafted the manuscript, visualization.; b Lecturer, Division of Preventive Dentistry, Faculty of Dentistry & Graduate School of Medical and Dental Sciences, Niigata University, Niigata, Japan. Conceptualization, methodology, formal analysis, investigation, data curation, reviewed and edited the manuscript, funding acquisition.; c Professor, Division of Oral Science for Health Promotion, Faculty of Dentistry & Graduate School of Medical and Dental Sciences, Niigata University, Niigata, Japan. Methodology, reviewed and edited the manuscript, project administration, funding acquisition.; d Professor, Division of Preventive Dentistry, Department of Oral Health Science, Graduate School of Dental Medicine, Hokkaido University, Hokkaido, Japan. Formal analysis, reviewed and edited the manuscript, funding acquisition.; e Assistant Professor, Division of Oral Science for Health Promotion, Faculty of Dentistry & Graduate School of Medical and Dental Sciences, Niigata University, Niigata, Japan. Reviewed and edited the manuscript, funding acquisition.; f Project Associate Professor, Department of Health Promotion Medicine, Niigata University Graduate School of Medical and Dental Sciences, Niigata, Japan. Investigation, data curation, reviewed and edited the manuscript, funding acquisition.; g Project Professor, Division of General Medicine, Uonuma Institute of Community Medicine, Niigata University Medical and Dental Hospital, Niigata, Japan. Reviewed and edited the manuscript, supervision, funding acquisition.; h Professor, Division of Clinical Nephrology and Rheumatology, Kidney Research Center, Niigata University Graduate School of Medical and Dental Sciences, Niigata, Japan. Reviewed and edited the manuscript, supervision, funding acquisition.; i Professor, Division of Preventive Dentistry, Faculty of Dentistry & Graduate School of Medical and Dental Sciences, Niigata University, Niigata, Japan. Reviewed and edited the manuscript, project administration.

**Keywords:** antibody level, atrial fibrilation, older adults, periodontitis, *Porphyromonas gingivalis*, older adults

## Abstract

**Purpose:**

To investigate the association between plasma antibody levels against *Porphyromonas gingivalis* (PG) and atrial fibrillation (AF) history in community-dwelling older individuals in Japan.

**Materials and Methods:**

This study was a subset of the Uonuma cohort study, including 3091 participants aged 60–79 years. Data were collected, including AF history as a dependent variable, plasma immunoglobulin G antibody levels against PG as an independent variable, and previously reported AF risk factors and demographic information as covariates.

**Results:**

The median age of the participants was 69 years. Of the 3091 participants, 1411 (45.6%) were men, and 56 (1.8%) had an AF history. AF prevalence was significantly higher in participants with higher antibody levels against PG than in those with lower antibody levels (3.0% vs 1.4%; p = 0.005). Multivariable logistic regression analysis showed that participants with higher antibody levels against PG had twofold higher odds of having AF (odds ratio = 2.13; 95% confidence interval = 1.23–3.69). Restricted cubic spline analysis indicated a nonlinear relationship between antibody levels against PG and AF history.

**Conclusion:**

Plasma antibody levels against PG were associated with AF history in community-dwelling older individuals in Japan.

The prevalence of noncommunicable diseases increases with age and with lifestyle changes. Worldwide, cardiovascular diseases (CVDs) account for half of all noncommunicable disease-related deaths,^5^ with an estimated 422.7 million prevalent cases and 17.92 million deaths globally in 2015.^36^ Atrial fibrillation (AF) is considered the most common cardiac arrhythmia associated with an increased risk of CVDs, such as cerebral infarction, myocardial infarction, and heart failure, with increased mortality rates.^21,32,37^ As the AF prevalence increases with age, it is predicted to increase further in the future.^17^ In the United States, the number of patients with AF is projected to increase approximately 2.5- to 3-fold from 2.3–5.1 million in the early 2000s to 5.6–15.9 million by 2050.^13,28,30^ In Japan, the number of patients with AF is expected to increase from 720,000 in 2005 to 1.03 million in 2050.^17^ Several studies have reported elevated levels of C-reactive protein, a systemic inflammation marker, in patients with AF, suggesting that systemic inflammation could lead to AF development and perpetuation.^3,10,27^ Fibrotic changes in the myocardium caused by the inflammatory cascade are the possible mechanism underlying systemic inflammation and AF, provoking further inflammation.^31^

Periodontitis is a chronic inflammatory disease induced by the host’s immune response to long-term periodontopathic bacterial infection. Periodontitis-induced local inflammation has been reported to be associated with various systemic diseases, including CVDs,^24,38^ such as arteriosclerosis^41^ and myocardial infarction,^42^ by inducing systemic inflammation. However, very few studies have investigated the association between AF and periodontitis. Therefore, the evidence is still insufficient.

*Porphyromonas gingivalis* (PG) is a major periodontopathic bacterium that plays an important role in periodontitis deterioration. PG could also be implicated in the development of various systemic diseases, including CVDs.^12^ Blood immunoglobulin G (IgG) antibody levels against PG are considered an acceptable surrogate marker for periodontal status based on clinical examination.^11,23,34^

This study aimed to investigate the association between periodontal conditions based on plasma antibody levels against PG and AF history in community-dwelling older individuals in Japan. The null hypothesis of this study was that plasma antibody levels against PG were not associated with AF history.

## Materials and Methods

### Study Population

This was a cross-sectional study and a subset of the Uonuma cohort study. The design and protocol of the Uonuma cohort study have been described in detail in previous studies.^19,20^ This study focused on Minamiuonuma City and Uonuma City. Briefly, the Uonuma cohort study is an ongoing study that targets individuals aged ≥40 years living in the Uonuma region of Niigata Prefecture, Japan. Health examination through medical interviews and blood collection as well as questionnaire administration were conducted in the baseline survey in 2012–2014. Considering the limited research resources and previous reports^13,17^ that have demonstrated AF prevalence to increase sharply in adults aged ≥60 years, this study targeted individuals aged 60–79 years who participated in the baseline survey of the Uonuma cohort study. Current smokers were excluded to eliminate their potential confounding effects on the association between periodontal condition and AF history.^22,33^ Individuals with edentulism were also excluded, since IgG antibodies against PG were unlikely to be detected for those who had lost all their teeth probably by the terminal stage of periodontal treatment.^2^ Additionally, individuals with missing data were excluded from statistical analyses.

### Measurement of Plasma IgG Antibody Levels against PG

Plasma samples were collected and stored at -80°C until further examination. Plasma IgG antibody levels against PG were measured using a chemical luminescent immunological automatic analyser (POCube, Toyobo; Osaka, Japan) with a reagent cartridge (Sunstar; Osaka, Japan). The readouts were then calculated to obtain specific plasma IgG levels against PG (U/ml). Gingipain, a proteolytic enzyme produced by PG, was targeted as the specific antigen.

### Data Collection

Data on AF history were obtained from the medical interviews (Table 1).

**Table 1:** Content of the medical interviews and questionnaire

Items	Content (answer)
**Medical interviews**	
Medical history	Atrial fibrillation, myocardial infarction, angina pectoris, and heart failure (yes, no)
Medication	Antihypertensive agents, hypoglycemic agents, and antidyslipidemic agents (yes, no)
Lifestyle	Drinking (daily, sometimes, rarely), amount of alcohol intake (<2 drinks, ≥2 and <4 drinks, ≥4 and <6 drinks, ≥6 drinks), smoking (Have you smoked at least 100 cigarettes in total since you were born? [yes, no]. If the answer is “yes”, do you still smoke now? [I still smoke, I have quit smoking])
**Questionnaire**	
Tooth number	How many teeth do you have? Crowned teeth and post-crown are counted as teeth. Implanted teeth are not counted. (fill in the number)

Participants’ body height (cm) and weight (kg) were measured, and their body mass index was calculated. Blood pressure was measured, plasma samples were collected, and the total and high-density lipoprotein cholesterol (mg/dl) and glycated hemoglobin levels were measured. Data on medication (including hypertension, dyslipidemia, and diabetes mellitus), medical histories (including myocardial infarction, angina pectoris, and heart failure), drinking status, and smoking status were obtained from the medical interviews. Data on the number of teeth were collected from a self-report questionnaire (Table 1).

AF risk factors, including obesity, systolic hypertension, diabetes mellitus, dyslipidemia, and excessive drinking, were defined as follows:^15,18,22^ obesity, body mass index ≥25 kg/m^2^; systolic hypertension, systolic blood pressure ≥140 mmHg or use of antihypertensive agents; dyslipidemia, non-high-density lipoprotein cholesterol levels of <130 or >189 mg/dl or use of antidyslipidemic agents; diabetes mellitus, glycated hemoglobin level as expressed in the National Glycohemoglobin Standardisation Program of ≥6.5% or use of hypoglycemic agents; excessive drinking, consuming four drinks (approximately 40 g alcohol) or more daily.

### Statistical Analyses

Based on the antibody levels against PG, participants were classified into two groups: the high group, which included participants with more than the third quartile of antibody levels against PG, and the low group, which included those with less than the third quartile. In the univariable analyses, quantitative variables were compared using the Mann-Whitney U-test, whereas qualitative variables were compared using the Χ^2^ test. Univariable logistic regression analysis was conducted using AF history (0: absence; 1: presence) as the dependent variable and antibody levels against PG (0: ≤third quartile; 1: >third quartile) as the independent variable (Model I). Moreover, three multivariable logistic regression models were constructed. Model II was adjusted for age and sex, and Model III was adjusted for age, sex, systolic blood pressure, and history of heart failure, for which significant differences in AF prevalence were observed in the univariable analyses. Model IV was adjusted for all AF risk factors. Furthermore, restricted cubic spline analysis with three knots (10%, 50%, and 90% percentiles) was performed in a logistic regression model to explore the potential nonlinear association between antibody levels against PG and AF history. The reference value was 2.64, which is the median value of antibody levels against PG in the low group. All statistical analyses were performed using Stata SE software version 14.0 (StataCorp LP; College Station, TX, USA). A p-value of <0.05 was considered statistically significant.

### Ethical Considerations

The study protocol was conducted in accordance with the Declaration of Helsinki and approved by the Ethics Committee of Niigata University (approval number: 2017-0071). Written informed consent was obtained from all participants before participation.

## Results

### Study Participants

**Fig 1:**
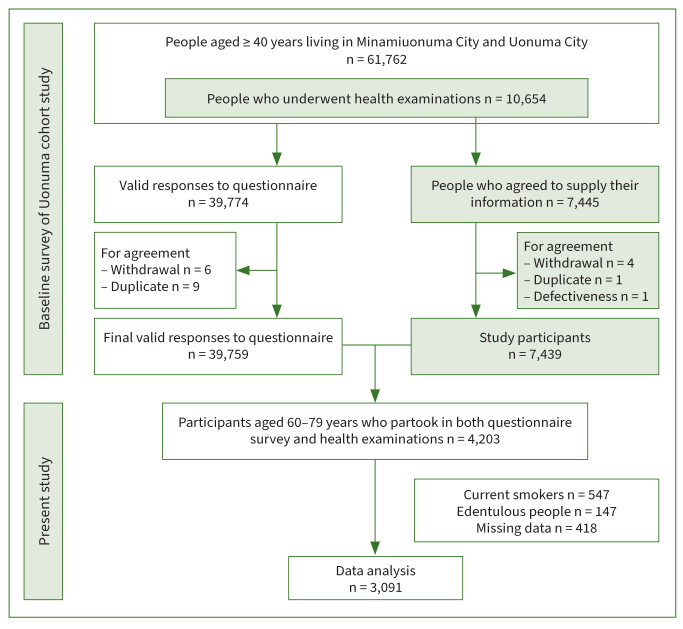
Flowchart of the study participant selection process.

Figure 1 presents a flowchart of the study participant selection process. At the baseline survey of the Uonuma cohort study, a questionnaire survey was conducted on 61,762 individuals living in the Uonuma region. Of these individuals, 39,759 (64.4%) reported valid responses, excluding those who withdrew their consent or were included in duplicate because of moving to another survey area during the survey period. Moreover, health examination through medical interviews and blood collection was conducted on 10,654 participants. Those who withdrew their consent, gave duplicate consent for multiple years, or gave defective consent were excluded from the study. As a result, 7439 participants agreed to provide their health information.

This study included 4203 participants aged 60–79 years who attended both the questionnaire survey and health examination. Of the 4203 participants, 1112 were excluded due to being active smokers (547 individuals), having edentulism (147 individuals), and having missing data (418 individuals). Thus, the final analysis was performed on 3091 participants.

### Characteristics of Participants According to the Antibody Level against PG

Table 2 shows a comparison of participants’ characteristics according to the antibody levels against PG. Of the 3091 participants, 56 (1.8%, 42 men) had an AF history. The overall median age was 69 years, and 1411 (45.6%) were men. An association was observed between higher antibody levels against PG and higher AF prevalence, lower median age, lower obesity prevalence, and higher prevalence of history of heart failure.

**Table 2:** Participants’ characteristics

Variables	Total	Antibody levels against PG		p-value
		Higher	Lower	
	n = 3091	n = 772	n = 2319	
AF history	56 (1.8)	23 (3.0)	33 (1.4)	0.005
Age	69 (65, 72)	68 (64, 72)	69 (65, 72)	0.031
Men	1411 (45.6)	354 (45.8)	1057 (45.6)	0.894
Obesity	654 (21.2)	139 (18.0)	515 (22.2)	0.013
Systolic hypertension	1603 (51.9)	393 (50.9)	1210 (52.2)	0.540
Dyslipidemia	1648 (53.3)	408 (52.8)	1240 (53.5)	0.764
Diabetes mellitus	280 (9.1)	71 (9.2)	209 (9.0)	0.877
History of myocardial infarction	27 (0.9)	6 (0.8)	21 (0.9)	0.740
History of angina pectoris	69 (2.2)	15 (1.9)	54 (2.3)	0.530
History of heart failure	6 (0.2)	4 (0.5)	2 (0.1)	0.018
Excessive drinking	182 (5.9)	55 (7.1)	127 (5.5)	0.092

Quantitative variables are shown as median (first quartile and third quartile), and qualitative variables are shown as number of participants (%). The Mann-Whitney U-test was used to compare quantitative variables, and the χ2 test was used to compare qualitative variables. AF: atrial fibrillation; PG: *Porphyromonas gingivalis*.

### Logistic Regression Analyses for AF History

The results of the univariable and multivariable logistic regression analyses for AF history are shown in Table 3. In all models, a statistically significant association was observed between higher antibody levels against PG and AF. The odds ratios (ORs) in each model were comparable, and in Model IV, participants with higher antibody levels against PG had more than twofold higher odds of having AF (OR = 2.13; 95% confidence interval [CI] = 1.23–3.69; p < 0.01). Male sex (OR = 2.66; 95% CI = 1.38–5.14; p < 0.005) and systolic hypertension (OR = 2.35; 95% CI = 1.23–4.49; p < 0.01) were associated with AF, respectively, although the decreases in ORs within a statistically significant level were observed as the number of independent variables increased. Age and history of heart failure were associated with AF in the univariable model (Model I). However, this association was not statistically significant in the multivariable models (Models II–IV). No statistically significant association was observed between other variables and AF in both univariable and multivariable models.

**Table 3:** Logistic regression analyses for AF history

Variables	Model I OR (95% CI)	Model II OR (95% CI)	Model III OR (95% CI)	Model IV OR (95% CI)
Higher antibody levels against PG	2.13 (1.24–3.65)^†^	2.18 (1.27–3.74)^†^	2.13 (1.24–3.68) ^†^	2.13 (1.23–3.69)^†^
Age	1.09 (1.03–1.15)^‡^	1.05 (1.00–1.11)	1.04 (0.99–1.10)	1.04 (0.99–1.10)
Men	3.65 (1.99–6.71)^§^	3.12 (1.65–5.91)^§^	2.72 (1.43–5.16) ^‡^	2.66 (1.38–5.14)^‡^
Obesity	1.50 (0.84–2.70)			1.26 (0.69–2.31)
Systolic hypertension	3.13 (1.67–5.84)^§^		2.47 (1.31–4.67) ^†^	2.35 (1.23–4.49)^†^
Dyslipidemia	1.36 (0.79–2.34)			1.25 (0.72–2.17)
Diabetes mellitus	1.69 (0.79–3.62)			1.19 (0.54–2.62)
History of angina pectoris	0.79 (0.11–5.82)			0.47 (0.06–3.60)
History of heart failure	11.02 (1.27–95.88)*		4.02 (0.44–36.40)	4.26 (0.44–40.89)
Excessive drinking	1.95 (0.82–4.61)			1.16 (0.47–2.86)

Model I was not adjusted for any variable. Model II was adjusted for age and sex. Model III was adjusted for age, sex, systolic hypertension, and history of heart failure. Model IV was adjusted for all AF risk factors. AF: atrial fibrillation; CI: confidence interval; OR: odds ratio; PG: *Porphyromonas gingivalis*; *p < 0.05; ^†^p < 0.01; ^‡^p < 0.005; ^§^p < 0.001.

### Restricted Cubic Spline Analysis

**Fig 2:**
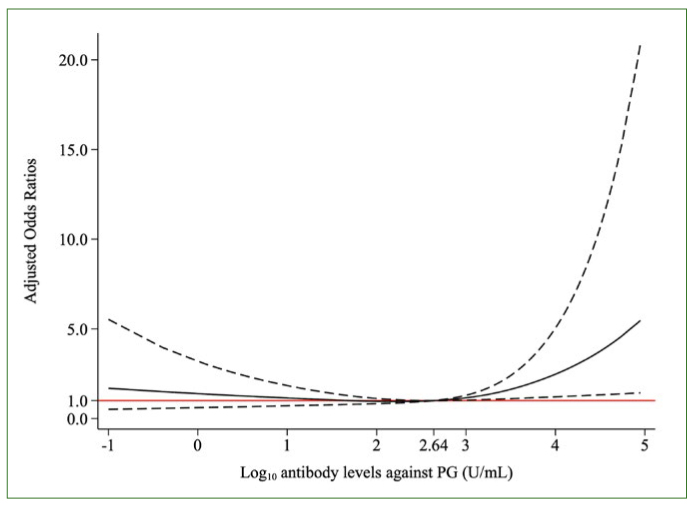
Restricted cubic spline showing the relationship between antibody levels against PG and AF history. The black solid line indicates adjusted odds ratios for AF history. The dashed lines indicate 95% confidence intervals. The red solid line is the reference line. The model was adjusted for age, sex, obesity, systolic hypertension, dyslipidemia, diabetes mellitus, history of angina pectoris, history of heart failure, and excessive drinking. AF: atrial fibrillation; PG: *Porphyromonas gingivalis*.

The restricted cubic spline analysis showed a nonlinear relationship between antibody levels against PG and AF history (Fig 2). An increased AF prevalence based on statistically significant ORs was confirmed markedly for the antibody levels exceeding the third quartile (3.35 U/ml), whereas statistically no significant increase for the lower antibody levels was observed.

## Discussion

This study showed a nonlinear relationship between antibody levels against PG and AF history. Participants with higher antibody levels against PG were approximately 2.1 times more likely to have an AF history than those with lower antibody levels, after adjustment for previously reported AF risk factors. These results suggest that deteriorating periodontal condition contributes to an increased risk of AF development independent of previously reported AF risk factors.

The association between periodontitis and AF has been reported in several previous studies. A population-based cohort study in Taiwan reported that the risk of AF development was increased by 31% in patients with periodontitis compared with those without periodontitis.^7^ Another study reported that patients with AF and periodontitis had a statistically significantly higher incidence of arrhythmic events and major adverse cardiac events than those without periodontitis.^16^ A previous study reported the effect of periodontitis on AF treatment outcomes,^29^ in which patients with higher antibody levels against PG type IV showed statistically significantly higher late recurrence rates after radiofrequency catheter ablation than those with lower antibody levels. Thus, periodontitis may have adverse effects on AF treatment outcomes. In this study, higher antibody levels against PG were statistically significantly associated with a higher risk of AF, even after adjusting for previously reported AF risk factors. These results suggest an association between periodontal condition and AF, and are consistent with those reported in previous studies, although the evaluation methods for periodontal disease vary.

Dental treatment and oral health behaviour have been reported to be associated with AF. For dental treatment, dental scaling at least once a year could reduce the risk of AF occurrence, and the protective effect of reducing AF occurrence was greater in individuals who underwent dental scaling more frequently.^8^ In terms of oral health behaviour, individuals who brushed their teeth three times or more a day had a lower risk of AF development than those who did 0–1 time a day.^6^ Furthermore, regular dental care was associated with a lower risk of AF development.^39^ These treatments and behaviour are fundamental measures for preventing periodontitis. Therefore, the results of these studies suggest that preventing periodontitis reduces the risk of AF development.

Inflammation plays an important role in the mechanism by which periodontitis affects AF development. Aarabi et al^1^ reported five fundamental mechanisms explaining the effect of oral inflammations, including periodontitis, on AF development: (I) low bacteremia leading to the invasion of oral bacteria into the cardiac atrium; (II) systemic inflammation caused by various types of inflammatory mediators leading to cardiac remodeling; (III) autoimmune response to molecular structures in the cardiac atrium due to the host immune response to oral pathogens; (IV) potentially arrhythmic effects through the activation of the autonomic nervous system initiated by oral inflammation; and (V) arrhythmic effects caused by bacterial proteins and toxins derived from oral pathogenic bacteria, such as PG. An animal experiment proved that periodontitis induced an inflammatory response in the atrial myocardium and caused changes in cardiomyocytes, such as hypertrophy and myolysis. This led to structural and electrophysiological changes in the cardiac atrium, increasing susceptibility to AF.^43^ This study did not clarify the mechanism by which periodontal condition affects AF development. However, since it evaluated the association between periodontal condition and AF based on plasma antibody levels against PG, which could be a biological marker reflecting the host immune response against PG, the results supported the inflammation-mediated mechanism reported in previous studies.

This study has several strengths. First, plasma IgG antibody levels against PG were measured to examine the association between periodontal condition and AF. Previous studies have used various classifications, such as the international classification of diseases, community periodontal index, and periodontal profile class, to define periodontitis.^7,16,39^ However, all these classifications are based on clinical periodontal probing. Clinical parameters, such as the depth of periodontal pockets, are measured to assess the local periodontal conditions. However, they are not direct measures of the systemic burden of the infection by oral bacteria.^4^ Therefore, the use of periodontal classifications based on these clinical parameters has been criticised when assessing the association between periodontitis and systemic diseases. Systemic antibody levels against periodontopathic bacteria, including PG, could be a more precise measure,^25^ because they reflect the host’s immune response to long-term infection by these bacteria. Second, this study determined a large number of antibody levels against PG. This study targeted more than 3000 older individuals and measured their plasma antibody levels. According to a pertinent literature survey, no previous study has examined the association between periodontitis and AF by measuring antibody levels against PG among thousands of community-dwelling older individuals in Japan.

This study has several limitations. First, this cross-sectional study could not ascertain the timepoint at which infection by PG was established and when AF developed. Therefore, the causal relationship between periodontal conditions and AF remains unclear, which is the major limitation. Second, AF data were obtained from medical interviews and thus depended on participants’ memory. Nevertheless, the incidence of AF history in the participants was 1.93%, 3.43%, 0.48%, and 1.43% for men in their 60s, men in their 70s, women in their 60s, and women in their 70s, respectively, and these rates are quite similar to those reported in the study conducted on approximately 630,000 people aged ≥40 years who underwent periodic health examinations.^17^ Therefore, the negative effect of using data from the medical interviews on the results of this study appears small. Third, plasma antibody levels against other periodontopathic bacteria such as *Aggregatibacter actinomycetemcomitans* (AA) and *Tannerella forsythia* (TF) were not measured in this study. According to a previous study that reported the distribution of oral bacteria in atherosclerotic plaques, however, PG was only identified in cardiac tissue, while AA and TF were present in multiple non-cardiac organs.^9^ This localisation suggests that PG can play an important role in cardiac diseases. Finally, this study did not clarify whether the effect of periodontal disease on AF was mediated by systemic inflammation, because data on systemic inflammation markers was not collected. The levels of serum inflammatory markers, such as C-reactive protein, and interleukin-6, were elevated in patients with periodontitis,^26,40^ and these markers were associated with AF risk.^14,35^ Thus, further longitudinal studies are needed to investigate the association between plasma antibody levels against PG and AF, in which AF is diagnosed based on an electrocardiogram, and systemic inflammation is assessed by serum inflammatory markers.

## Conclusion

Plasma antibody levels against PG were associated with AF history in community-dwelling older individuals in Japan. Further prospective studies are needed to determine the temporal association between plasma antibody levels against PG and AF.
